# Testing the stability of ‘Default’ motor and auditory-perceptual rhythms–A replication failure dataset

**DOI:** 10.1016/j.dib.2020.106044

**Published:** 2020-07-20

**Authors:** Anat Kliger Amrani, Elana Zion Golumbic

**Affiliations:** The Gonda Brain Research Center, Bar Ilan University, Ramat Gan, Israel

**Keywords:** Motor rhythms, Auditory perception, Finger tapping, Synchronization, Auditory-Motor interactions

## Abstract

Several studies have found that the motor rhythms that individuals produce spontaneously, for example during finger tapping, clapping or walking, are also rated perceptually as ‘very comfortable’ to listen to. This motivated proposal of the *Preferred Period Hypothesis*, suggesting that individuals have a characteristic preferred rhythm, that generalizes across perception and production.

However, some of the experimental procedures used previously raise two methodological concerns: First, in many of these studies, the rhythms used for assessment of participants’ Perceptual Preferred Tempo (PPT) were tailored specifically around each participant's personal Spontaneous Motor Tempo (SMT). This may have biased results toward the central rhythm used, artificially increasing the similarity between spontaneous motor and auditory perceptual preferences. Second, a key prediction of the *Preferred Period Hypothesis* is that the same default rhythms are repeatedly found within-subject. However, measures of consistency are seldom reported, and increased within-subject variability has sometimes been used to exclude participants.

The current study was an attempt to replicate reports of a correspondence between motor and perceptual rhythms, and closely followed previous experimental protocols by conducting three tasks: SMT was evaluated by instructing participants to tap ‘at their most comfortable rate’; PPT was assessed by asking participants to rate a 10 different rhythms according to how ‘comfortable’ they were; and motor-replication of rhythms was assessed using a Synchronization-Continuation task, over a wide range of rhythms.

However, in contrast to previous studies, for all participants we use the same 10 perceptual rhythms in both the PPT and Synchronization-Continuation task, irrespective of their SMT. Moreover, we assessed and report measures of within- and between-trial consistency, in order to evaluate whether participants gave similar rating and produced similar motor rhythms across multiple sessions throughout the experiment.

The data presented here fail to show any correlation between motor and perceptual preferences, nor do they support improved synchronization-continuation performance near an individual's so-called SMT or PPT. Rather, they demonstrate substantial within-subject variability in the spontaneous motor rhythms produced across repeated sessions, as well as their subjective rating of perceived rhythms. This report accompanies our article *“Spontaneous and Stimulus-Driven Rhythmic Behaviors in ADHD Adults and Controls”*[1], and provided motivation and insight for modifying the procedures used for SMT and PPT evaluation, and their interpretation.

Specifications Table

**Table:** 

Subject	Behavioural Neuroscience
Specific subject area	Testing the correspondence between spontaneous motor rhythms, rhythmic perceptual preferences and auditory-motor synchronization
Type of data	Graphs Figures Raw Data + code
How data were acquired	Participants were seated comfortably in a sound attenuated booth, and heard sounds through headphones (Sennheiser HD 280 pro). The experiment was programmed and controlled using PsychoPy software (www.psychopy.org). Finger taps were recorded using a custom-made tapper based on an electro-optic sensor.
Data format	Raw data files ('.mat') and code for analysis ('.m')
Parameters for data collection	Participants were recruited from a specific age group (20–28 years old), and were not diagnosed with any neurological or psychiatric clinical condition.
Description of data collection	Data was collected in a sound attenuated booth. Finger taps were recorded using a custom-made tapper based on an electro-optic sensor. Auditory stimuli were prepared using Audacity and Matlab (Mathworks) and heard through headphones (Sennheiser HD 280 pro). The experiment was programmed and controlled using PsychoPy software (www.psychopy.org).
Data source location	City: Ramat Gan Country: Israel
Data accessibility	Repository name: Center for Open Science, Open Science Framwork (OSF) Direct URL to data: https://osf.io/h3yut/
Related research article	Authors: Anat Kliger Amrani and Elana Zion Golumbic Title: Spontaneous and Stimulus-Driven Rhythmic Behaviors in ADHD Adults and Controls Journal: Neuropsychologia (under revision)

Value of the Data

These data constitute a failure to replicate previous reports of a correspondence between motor and perceptual “most comfortable” rhythms [2], [3], [4], [5], [6], [7], [8]. They reveal substantial variability in spontaneous rhythmic behavior – both within and across modalities - which is often overlooked when reporting only summary metrics (means). The data are useful for assessing the degree to which individuals have a single, and consistent, default rhythm that generalizes across perception and production.
In contrast to previous studies, assessment of perceptual rhythmic preferences (PPT) in the current experimental design was not tailored around each individual's spontaneous motor rhythm (SMT). Comparison of this dataset to data obtained in previous studies will be useful for researchers interested in evaluating the generalization of rhythmic preferences across modalities and their within-subject consistency over time.
These data are of value to neuroscientists and cognitive psychologists interested in the nature of rhythmic behaviours and their underlying neural mechanisms, as well as for clinicians involved in studying and treating different forms of motor and language deficits (e.g. Parkinson's disease, dyslexia, ADHD etc.).

## 1 Data description

**Fig. 1:**
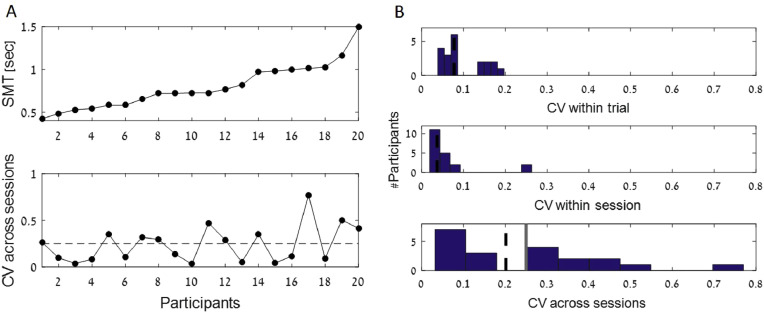
Spontaneous Motor Tapping results A) Median SMT values (top) and consistency across sessions (bottom) for individual participants, in ascending order of mean SMT. The horizontal dashed line indicates the cutoff of CV_across___sessions_ = 0.25, used in previous studies to exclude participants who had inconsistent SMTs across sessions. B) Distribution of CV_within_trial_ CV (top), CV_within_session_ (middle) and CV_across_sessions_ (bottom) across all participants. The dashed black line represents the group median and the gray line indicates the cutoff of CV_across___sessions_ = 0.25, shown also in A.

**Fig. 2:**
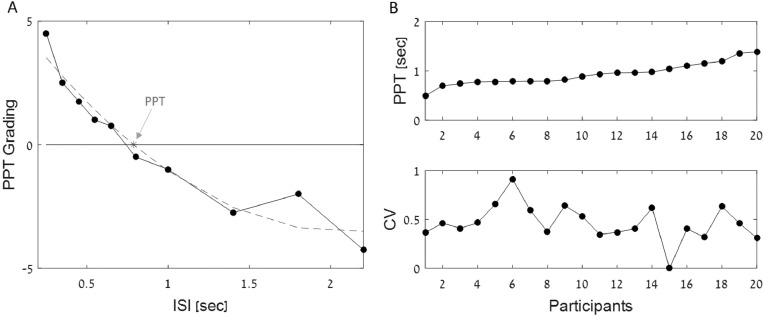
Preferred Auditory Perceptual Tempo results. A) Example from a single participant of the procedure to estimate PPT from the average gradings of 10 different rhythms (black dots) on a 10-point scale from −5 (too slow) to +5 (too fast), with 0 being “most comfortable”. The dashed gray line indicates the best polynomial fit, and the crossing-point indicated by a gray asterisk is the estimated PPT. B) Distribution of PPT values (top) and consistency (CV, bottom) for all participants, in ascending order of mean PPT.

**Fig. 3:**
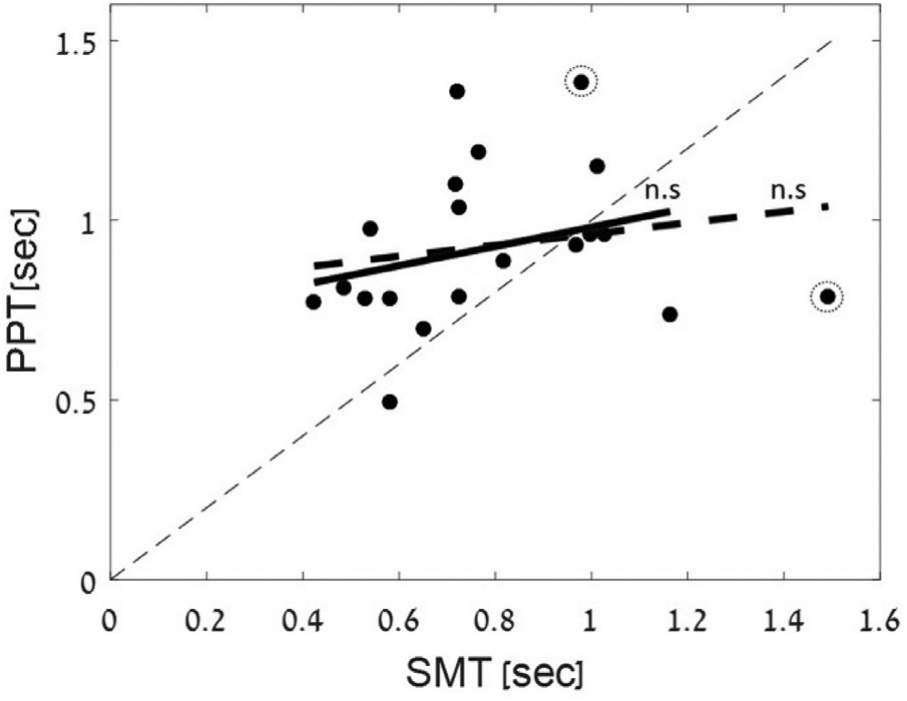
Relationship between SMT and PPT. Linear regression analysis testing the correspondence between the median SMT and PPT values obtained for each participant black dots. Neither the regular regression (dashed thick line) or robust regression (solid line; outliers marked in dashed circles) yielded significant results, indicating no correlation between the two measures. The thin dashed thin line is the diagonal unity line.

Fig. 1, Fig. 2, Fig. 3 describe the range and variability of Spontaneous Motor Tempo (SMT) and Preferred Perceptual Tempo (PPT), and the relationship between them.

Median SMT values across participants ranged between 0.4–1.5 s ITIs (median 0.73, Fig. 1A, upper panel). When quantifying SMT consistency across sessions though, we find that half of the participants (10/20) had a CV_across_sessions_ > 0.25 (Fig. 1A and 1B, lower panel), which is the threshold used previously to exclude participants who showed inconsistent spontaneous tapping [4]. Tapping CV_within_trial_ and CV_across_trials_ were more consistent (Fig. 1B, upper and middle panels).

PPT was evaluated for each participant using a polynomial fit of their average ratings of 10 different rhythms and extracting the zero-crossing point, which corresponded to the ‘most comfortable’ rhythm (Fig. 2A). PPT values and consistency across participants are shown in Fig. 2B.

Fig. 3 show the relationship between median SMT and PPT estimated for each participant. Linear regression analysis revealed no significant correlation between the two measures (*r* = 0.18, *p* > 0.4, dashed thick line; robust correlation: *r* = 0.27 *p* = 0.26, solid line, outliers marked in dashed circles).

**Fig. 4:**
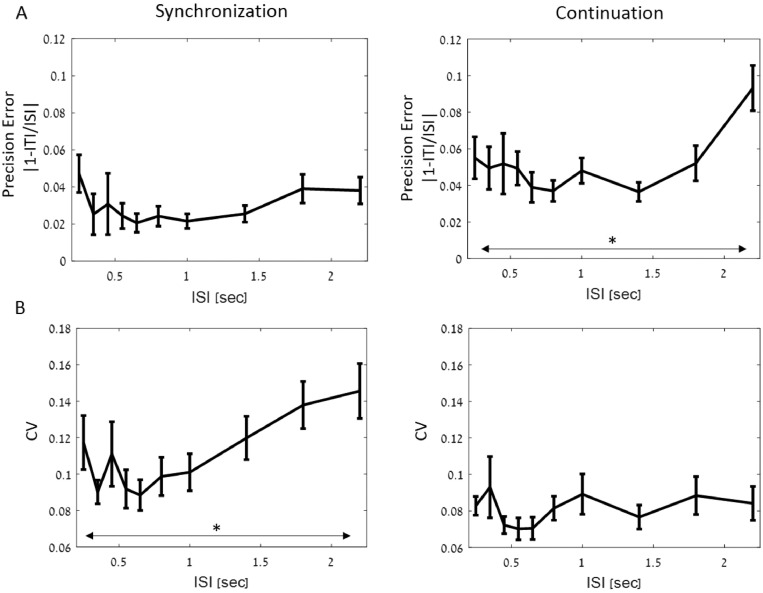
Synchronization and Continuation results. A) Mean tapping precision error for all tempi during synchronization (left panel) and continuation (right panel) task. The main effect of tempo was significant only in the continuation tasks. B) same as A) for CV_within_trial_, indicating degree of tapping isochrony. Here there was a main effect of tempo during the synchronization but not continuation task. Error bars depict SEM.

**Fig. 5:**
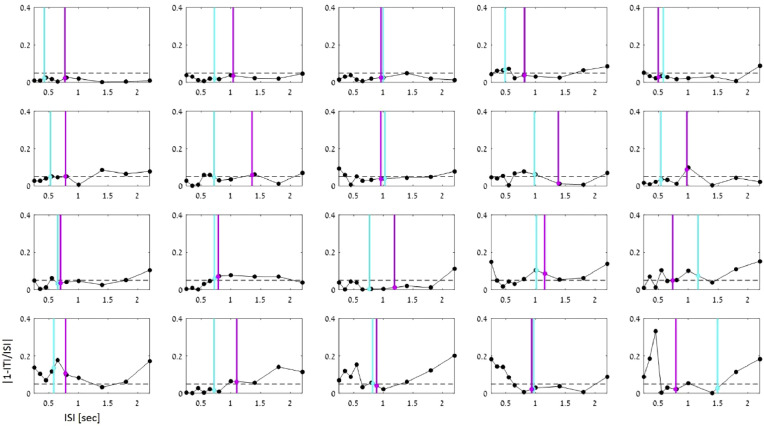
Continuation tapping precision-error for all participants. Subplots are ordered according to the degree of variability in precision errors across tempi. Each participant's median SMT and PPT are indicated by the cyan and magenta lines, respectively (the precision-error at SMT/PPT was estimated based on linear interpolation of the two nearest tempi, and is marked with a circle of the same color).

**Fig. 6:**
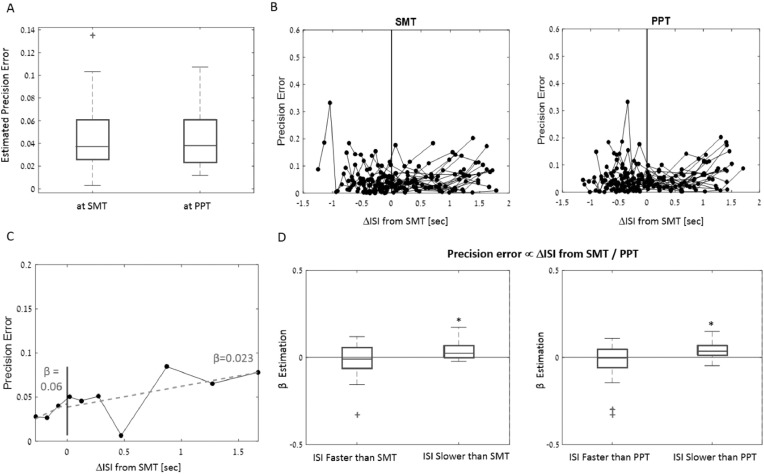
Precision error as a function of the distance from SMT / PPT: A) Continuation precision error estimated at each participants’ median SMT and PPT. Box plots depict the median and the 25/75th percentiles. Outliers are indicated by the + sign, (values are considered outliers if they are > 1.5 times the interquartile range from the top or bottom of the box). B) Precision error across tempi aligned relative to each participant's individual SMT and PPT. No apparent U-shape is observed, which would have suggested that performance is better near ones SMT/PPT. C) Example of the linear regression procedure applied to one example participant. A linear fit was performed separately for tempi faster (left) and slower (right) than the participants SMT, and slope values β were extracted for each side. D) Distribution of the estimated β slope values across all participants, showed separately for the analyses conducted relative to the SMT (left) and PPT (right). Box plots depict the group-median 25/75th percentiles. Outliers are indicated by the + sign. Precision error increased consistently for rhythms slower than both the SMT and PPT, but no consistent relationship was found for faster rhythms. This pattern is inconsistent with the notion that performance is optimal near one's SMT/PPT.

Figs. 4–6 present the results in the synchronization-continuation task, and their relationship to individual preferred rhythms (SMT and PPT).

Fig. 4 shows the tapping precision-error and degree of isochrony (CV_within_trial)_ for all 10 tempi tested, separately for the synchronization and continuation stages. A repeated-measures ANOVA revealed no differences in precision-error across tempi during the synchronization stage [F(9,19) = 1.45, *p* = 0.16; Fig. 4A left], but the degree of isochrony CV_within_trial_ did show a main effect of tempo during synchronization [F(9,19) = 4.69, *p* < 10^−4;^ 4B left], reflecting reduced isochrony at the two slowest rhythms. For continuation tapping there was a main effect of tempo for precision-error, also stemming from increased errors for the two slowest rhythms [F(9,19) = 3.41, *p* = 0.0007; Fig. 4A right], however the CV_within_trial_ was not significantly different across tempi [F(9,19) = 1.31, *p* = 0.23; 4B right].

Fig. 5 shows individual-level data for all participants, of the average tapping precision error during the continuation stage for all tempi (performance was near-ceiling during synchronization), with their individual SMT and PPTs indicated as well. The correspondence between continuation-performance and individual SMT/PPT is quantified in Fig. 6. *Re*-aligning the tapping precision-errors to one's SMT / PPT showed no clear U-shape, but only a minor rise towards the slower range (Fig. 6B). Linear estimation of the relationship between precision-error at each tempo as a function of its distance from an individual's SMT or PPT confirmed that this was the case only for slower rhythms (significant positive slope, sign test, *p* < 0.042 for both SMT and PPT alignments), but not for faster rhythms (*p* > 0.8; Fig. 6D).

## 2 Experimental design, materials, and methods

### 2.1 Experimental design

***Participants***: The experiment included 20 participants (10 women, age range 21–28, mean 24, 2 left handed). *N* = 20 participants were tested, after giving written informed consent. None of the participants were diagnosed with ADHD, or reported any other neurological or psychiatric clinical condition.

***Experimental Apparatus:*** Participants were seated comfortably in a sound attenuated booth, and heard sounds through headphones (Sennheiser HD 280 pro). The experiment was programmed and controlled using PsychoPy software (www.psychopy.org). Finger taps were recorded using a custom-made tapper based on an electro-optic sensor.

**Fig. 7:**
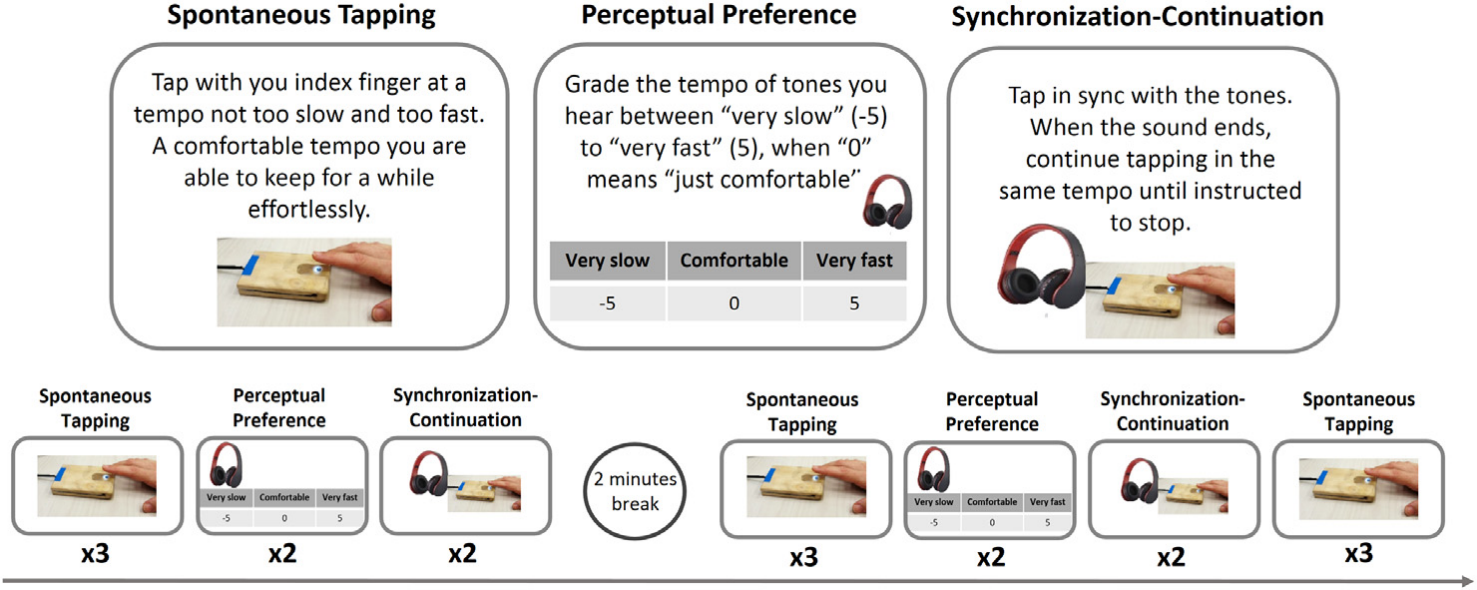
Experimental design. Top: The three tasks performed during the experiment. Bottom: Time line of performing each task and repetition across sessions.

***Procedures and Stimuli:*** All auditory stimuli were prepared using Audacity and Matlab (Mathworks), and consisted of repetitions of pure tones (440 Hz, 30 ms with ±5 ms ramp up/down), presented at different rates. The experiment consisted of three tasks, performed in interleaved order as shown in Fig. 7.

***Spontaneous Tapping Task:*** Participants tapped with their preferred index finger at a constant rhythm for 30 taps, following the instruction “Tap at your most comfortable rate, not too slow and not too fast, but at a rhythm that feels just right”. Participants repeated the spontaneous tapping task in three separate sessions throughout the experiment, with each session containing three consecutive tapping trials. These repetitions were used to test for tapping consistencies at different time-scales.

***Auditory Perceptual Preference Task****:* Participants listened to sequences of tones at 10 different tempi (250, 350, 450, 550, 650, 800, 1000, 1400, 1800, 2200 ms ISI; 10 s long trials), presented in random order. They were asked to grade each tempo on a scale between−5 being very slow to +5 being very fast, when the grade 0 means comfortable. Participants repeated the PPT task in two separate sessions throughout the experiment, with each session containing two consecutive trials in which tempi were presented in a randomized order.

***Synchronization-Continuation Task:*** Participants heard a sequence of 30 tones (but up to 30 s) at a particular tempo and tapped along with them (Synchronization stage). Then a stop sign appeared for 1.5 s, after which they were instructed to reproduce the previous tempo and tap the same amount of taps as they heard (Continuation stage), until stopped automatically. No feedback was given regarding the temporal accuracy of the tapping. Ten different tempi were used, presented in random order (ISIs: 250, 350, 450, 550, 650, 800, 1000, 1400, 1800 and 2200 ms). Participants performed the Synchronization-Continuation task in two separate sessions throughout the experiment, and each tempo was repeated twice in each session.

### 2.2 Data analysis

#### SMT

**Fig. 8:**
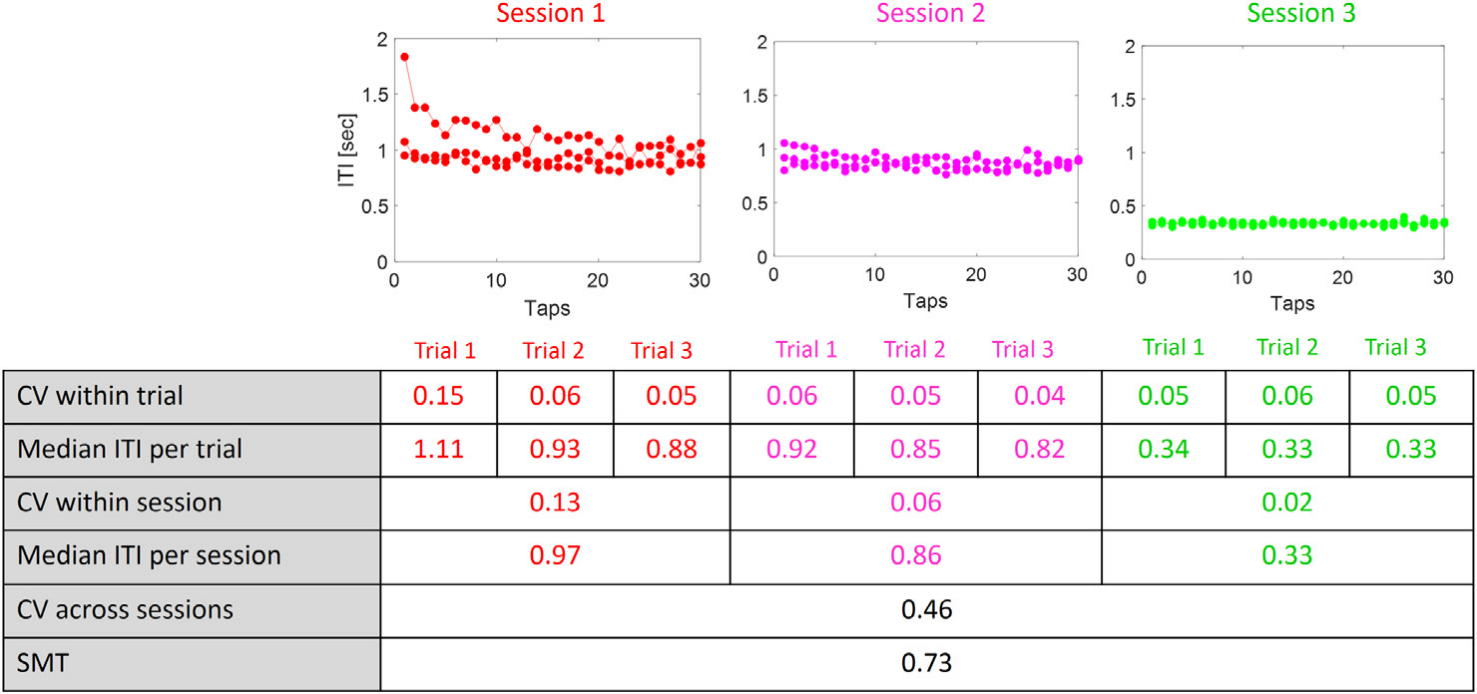
Analysis of Spontaneous Tapping Behavior - example from one participant. *Top:* Tapping ITIs in single trials, across all three sessions of the SMT task (three trials per session). *Bottom:* The central (median, mean) and consistency (CV) metrics derived from tapping ITIs, within and across session, to characterize different aspects of spontaneous tapping.

To quantify spontaneous tapping behavior, we assessed both central SMT measures (mean and median values), as well as consistency measures, within and across trials. All calculations were derived from the Inter-Tap-Intervals (ITIs) in individual trials, and are illustrated in Fig. 8. All taps were included in the analysis, without excluding initial taps or extremely long or short ITIs, since we regard any such variability as an integral part of tapping behavior.

Consistency measures were all based on the Coefficient of Variation ($CV=\;\frac{std}{mean})$, to avoid biases due to differences in tempo and allow comparability across tempi. We specifically calculated the following consistency metrics for each participant:

Within-trial tapping consistency (CV_within_trial_): Represents how isochronous the tapping was within a given trial. This is calculated using the ITIs across all ten taps in a given trial (Fig. 8, row 1).

$$CV_{within\_trial}=\frac{std\left(ITI_{within\_trial}\right)}{mean\left(ITI_{within\_trial}\right)}$$

Within-session tapping consistency (CV_within_session_): Represents whether participant replicate the same median rhythm in consecutive trials within a session. This is calculated using the median ITI values from the three trials within each session (Fig. 8, rows 2,3):

$$CV_{within\_session}=\frac{std\left(Median\_ITI_{per\_trial}\right)}{mean\left(Median\_ITI_{per\_trial}\right)}$$

Across-session tapping consistency (CV_across_session_): Represents whether participant replicate the same median rhythm in different sessions throughout the experiment. This is calculated using the median ITI values from the three sessions (Fig. 8, rows 4,5).

$$CV_{across\_session}=\frac{std\left(Median\_ITI_{per\_session}\right)}{mean\left(Median\_ITI_{per\_session}\right)}$$

Finally, the average Spontaneous Motor Tempo (SMT) was calculated by averaging the median ITIs across all three sessions (Fig. 8, row 6).

#### PPT

To quantify perceptual rhythmic preferences, we used the grades that participants gave to the 10 tempi they listened to. For each tempo all four grades were averaged, and a polynomial fit was created based on the average grades for all tempi. The zero-crossing point of that curve indicates the tempo regarded as most comfortable, hence was termed the Preferred Perceptual Tempo (PPT). PPT Consistency was calculated by taking the median of CV values for the four sessions of each tempo.

#### SMT-PPT correlation

We performed a linear regression analysis to evaluate the correspondence between the median SMT and PPT values obtained for each participant, fitting the data to a linear model $y=\beta x+\beta_{0}$. The strength of the correspondence was assessed statistically using the r-value associated with the goodness of fit of the model. To remove potential biases due to outliers, the analysis was also repeated using robust linear regression [9].

#### Synchronization-Continuation

Tapping precision in the Synchronization-Continuation task was evaluated by calculating the ratio between the mean ITI produced in each trial and the prescribed ISI of the stimulus (ITI/ISI) as well as the precision error: |1−ITI/ISI|. We also estimated how isochronous the tapping was by calculating the CVwithin_trial (similar to the procedure described above for spontaneous tapping). These were calculated separately for the Synchronization and Continuation stages. We tested if tapping precision-error or tapping isochrony (CVwithin_trial) were modulated by tempo or group using a repeated-measures ANOVA with the factor Tempo (10-levels).

#### Modulation of synchronization-continuation performance by SMT/PPT

Last, we tested the prediction of the Preferred Period Hypothesis that tapping in the Synchronization-Continuation task is better near ones’ SMT/PPT, using a nested linear regression analysis aimed at evaluating whether performance at each tempo was modulated its distance from the participants SMT or PPT (separate analyses). This analysis focused on the precision-error values in the Continuation stage, since performance during Synchronization was near ceiling. For each participant we estimated two separate regression-lines, for rates either faster and slower than the SMT/PPT, and extracted the slopes (β) estimated for each participant from each of the regressions (see illustration of the procedure in Fig. 6C). Next, we tested whether the slopes estimated from each regression shared similar signs and if their distribution differed significantly from a null distribution around zero using a sign-test. According to the predictions of the Preferred Period Hypothesis, we would expect to find significantly negative slopes for rhythms faster than ones SMT/PPT, and positive slopes for rhythms slower than ones SMT/PPT.

## Ethics statement

The study was approved by the IRB committee at Bar Ilan University, and all participants provided written informed consent prior to the start of the experiment.

## Declaration of Competing Interest

The authors declare that they have no known competing financial interests or personal relationships which have, or could be perceived to have, influenced the work reported in this article.
